# Integration of machine learning with complex industrial mining systems for reduced energy consumption

**DOI:** 10.3389/frai.2022.938641

**Published:** 2022-07-27

**Authors:** Michael David Harmse, Jean Herman van Laar, Wiehan Adriaan Pelser, Cornelius Stephanus Lodewyk Schutte

**Affiliations:** Department of Industrial Engineering, Stellenbosch University, Stellenbosch, South Africa

**Keywords:** integrated dynamic control, energy management, real applications in engineering, deep-level mining, artificial intelligence, predictive modeling, optimization, machine learning

## Abstract

The deep-level mining industry is experiencing narrowing profit margins due to increasing operating costs and decreasing production. The industry is known for its lack of dynamic control across complex integrated systems running deep underground, making IoT technologies difficult to implement. An important integrated system in a typical underground mine is the refrigeration-ventilation system. In practice, the two systems are still controlled independently, often due to a lack of continuous measurements. However, their integrated effects ultimately affect energy usage and production. This study develops and compares various machine learning prediction techniques to predict the integrated behavior of a key component operating on the boundary of the refrigeration-ventilation system, while also addressing the lack of continuous measurements. The component lacks sensors and the developed industrial machine learning models negate the effect thereof using integrated control. The predictive models are compared based on accuracy, prediction time, as well as the amount of data required to obtain the required level of accuracy. The “Support Vector Machines” method achieved the lowest average error (1.97%), but the “Artificial Neural Network” method is more robust (with a maximum percentage error of 12.90%). A potential energy saving of 215 kW or 2.9% of the ventilation and refrigeration system, equivalent to R1.33-million per annum ($82 900^1^) is achievable using the “Support Vector Machines” method.

## 1. Introduction

**Figure 1:**
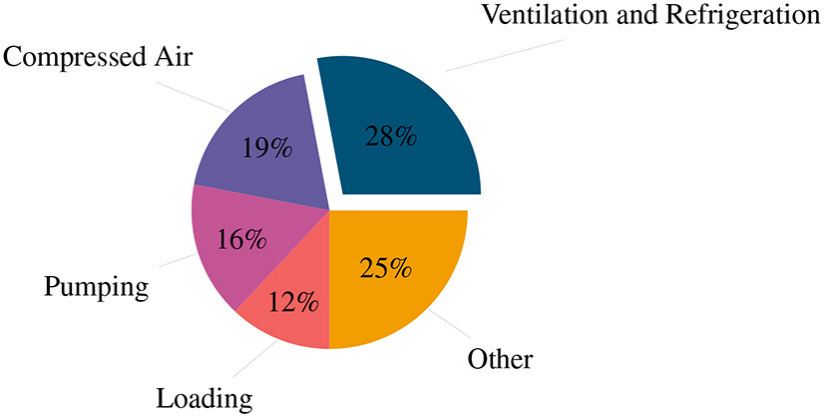
Energy distribution of a typical deep-level mine.

The deep-level mining industry is experiencing narrowing profit margins due to increasing operating costs and decreasing production (Ruffini, 2010; Lane et al., 2015; Neingo and Tholana, 2016). Electricity consumption is the second largest operating cost (after labor) with refrigeration and ventilation being the largest combined energy consumer in the industry, deep-level mining as depicted in Figure 1 (Cilliers et al., 2016; Crawford et al., 2019). Optimizing the refrigeration and ventilation system will result in a reduced energy consumption and an increased profit margin.

Ventilation is required to extract heat from the underground environment and ensure that working conditions are within legal limits (Nel et al., 2018; South Africa, 2018). However, for mines with high virgin rock temperatures and large depths below surface, refrigeration is required to maintain low temperatures. This increases the heat extraction capacity of the air going underground (Peach et al., 2018).

The refrigeration system utilizes water as a heat exchange medium between the air going underground and the ambient air, facilitating heat rejection into the ambient air. The outlet water temperature set-point is typically a static value based on “worst-case” design conditions and does not account for real-time ambient conditions (Maré, 2017; Peach et al., 2018). The result is an oversupply of cooling to the air going underground, resulting in energy wastage (Nell et al., 2019). A varying set-point will enable the system to reduce the cooling required and consequently reduce the energy consumption.

Varying the set-point requires a complex integrated control philosophy. Characteristic systems are required to model the actual system and predict parameters to account for changes in the system. The characteristic system can then be used as a predictive model in a dynamic control philosophy (Hasan et al., 2013; Chu et al., 2014). This has not been done in the deep-level mining industry due to the lack of sensor infrastructure and the large and complex integrated nature of the systems in these mines (Prinsloo et al., 2019).

The lack of sensor infrastructure in mining prevents the implementation of semi-empirical and mathematical-based control and predictive philosophies (Nell et al., 2019; Prinsloo et al., 2019). The lack of sensors result in data points that lack features (a measureable piece of data i.e., a column in a dataset). Characteristic models are thus required to replicate a system from feature-missing data. Fuzzy systems have been used in other applications to overcome a shortage of data points to develop relationships between dependent and independent variables but not to overcome missing features within a dataset (Savic and Pedrycz, 1991).

Machine Learning (ML) predictive techniques have the ability to characterize systems with little data and, although the number of features affect the accuracy, predictive techniques can characterize systems with little features (Al-Mukhtar et al., 2020). Three of the most commonly used ML predictive techniques are (Alanazi et al., 2017; Hassan et al., 2021):

1. *Artificial Neural Networks:* Artificial Neural Networks (ANN) have been used in various characteristic models (Al-Mukhtar et al., 2020; Teymen and Mengüç, 2020; O'Kelly and Soltani, 2021). These models show good accuracy compared to regression models where all the features are present (O'Kelly and Soltani, 2021). However, these models were implemented on systems with all the features accurately measured and have not been implemented on a system with lacking feature sensors.
2. *Support Vector Machines:* Support Vector Machines (SVM) have a specific algorithm for regression, which is called Support Vector Regression (SVR). SVRs have been compared to Response Surface Methodologies for predictive applications (Mia and Dhar, 2019). Similarly, these models have been used for prediction purposes (Temeng et al., 2020), but lack implementations on systems lacking features within data points.
3. *Nearest Neighbor Regression:* The k-Nearest Neighbor (k-NN) algorithm is commonly used for classification (Demidova and Sokolova, 2021; Komatwar and Kokare, 2021) but can also be used in regression applications (Papadopoulos et al., 2011). However, the focus of k-NN implementations has been on complete datasets (not lacking features).

A literature survey was conducted to evaluate the existing solutions with regards to integrated control within the mining industry with limited sensor infrastructure—a summary of the literature review can be seen in Table 1. The purpose of the table is to identify the gaps in previous research. The fields that form the comparison for the literature are:

- Machine Learning:
  - Predictive Methods: Were predictive ML methods utilized in the study?
  - Limited Features: Did the data used to develop models lack features?
- Deep-level Mining Industry:
  - Integrated Control: Did the studies implement control on multiple dependent systems within the deep-level mining industry?
  - Enhanced System Control: Did the studies implement dynamic control on systems within the deep-level mining industry?

**Table 1:** State-of-the-art matrix indicating gaps in previous research.

**Sources**	**Machine learning**		**Deep-level mining industry**		**Comments**
	**Predictive methods**	**Limited features**	**Integrated control**	**Enhanced system control**	
Youssefi et al. (2009), Papadopoulos et al. (2011), Al-Mukhtar et al. (2020), and O'Kelly and Soltani (2021)	✔	✘	✘	✘	Utilized ANN or k-NN for prediction on full-feature datasets outside of the mining industry. Obtained R^2^ values above 0.8 on all datasets.
Wang et al. (2019), Harmse (2021), and Harmse et al. (2022)	✔	✘	✘	✔	Utilized ANN for prediction on full-feature datasets on a single system within the mining industry, obtaining errors less than 5%.
Hasan et al. (2013), Hasan et al. (2014), and Hasan and Twala (2016)	✔	✔	✘	✔	Utilized limited feature datasets on an independent system within the mining industry. ANN and SVM obtaining higher accuracy for classification compared to “Naïve Bayes” “Classifier” and “Decision Trees.”
Cilliers et al. (2016), Maré (2017), Crawford et al. (2019), and Pretorius et al. (2019)	✘	✘	✘	✔	Improved control on independent systems within the mining industry, through simulation.
Arndt (2000) and Du Plessis et al. (2015)	✘	✘	✔	✔	Improved control on multiple dependent systems with full-feature monitoring within the mining industry, through simulation.


Appropriate keywords from each research field (such as predictive control, Machine Learning, limited data, deep-level mining, etc.) were identified and used individually and in a combination to identify relevant research within various databases (such as Springer and ScienceDirect). The relevant research was identified and summarized in Table 1, Further, Table 1 indicates relevant research fields that were not addressed within the reviewed studies.

As highlighted in Table 1, previous studies have utilized predictive ML methods to overcome the lack of features within the deep-level mining industry for enhanced system control. However, these models have not been implemented on the complex integrated systems within the mining industry. Further, an evaluation of these methods will benefit the industry to reduce wastage by implementing integrated dynamic control on large energy-consuming systems, such as the refrigeration- and ventilation system (Prinsloo et al., 2019).

This study provides a comparison of ML predictive techniques through an application in the deep-level mining industry. The comparison will serve to identify the best technique to utilize in the implementation of an integrated dynamic control philosophy on the refrigeration system. The comparison will be focused on the deep-level mining industry to accommodate for the shortcomings of the industry, mainly concerning the lack of feature sensors.

## 2. Prediction model development

### 2.1. Application

A Bulk Air Cooler (BAC) uses cold water to remove heat from the air supplied to the underground environment and acts as the interface between the refrigeration and ventilation systems. Refrigeration plants cool the water in a closed loop system. The BAC discharge air is the inlet of the ventilation system. The ventilation system is a static, stable system and only varies with the inlet temperature (BAC discharge temperature).

**Figure 2:**
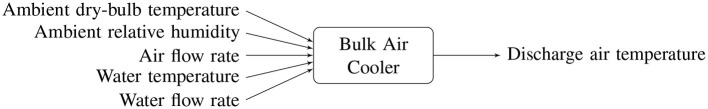
Application layout depicting parameters affecting the discharge air temperature.

The BAC discharge air temperature is dependent on the inlet parameters. The inlet and outlet parameters are depicted in Figure 2. The adjustable inlet parameters are air flow rate, water temperature and water flow rate. However, the air flow rate is determined through the air flow requirement of the shaft and the water flow rate cannot be changed due to the pumping configuration. Thus, the only available variable for control is the water temperature.

A static discharge temperature for the BAC is required, due to the static, stable nature of the ventilation system. To achieve this, the inlet parameters (water temperature) need to vary based on the other inlet parameters. However, in the present control strategy, the water temperature is static. The water temperature set-point can be determined from the other varying inlet parameters and the required discharge temperature by using ML techniques.

### 2.2. Data

Characterizing the BAC performance from historic data will allow accurate control. However, the ambient relative humidity is not being measured and relationships between the other parameters are required to characterize the BAC. Half-hourly data was obtained for four summer months to represent the BAC operation. The data was filtered and time periods were excluded that experienced irregular operation (for example, production stoppages, employee strikes, etc.). A total of 1 655 data points were used to characterize the BAC.

**Figure 3:**
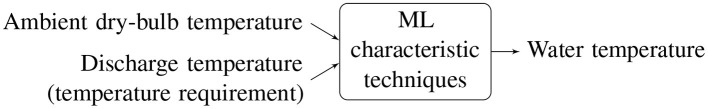
Input variables and output variable of the various characteristic models.

The various ML characteristic techniques determine the relationships according to their design regression characteristic. These relationships are determined from the filtered data and then used to determine the water temperature set-point. Due to a lack of sensors, only 2 variables are used as inputs. The same dataset structure, visualized in Figure 3, is applied to the various characteristic techniques.

### 2.3. Process control flow

**Figure 4:**
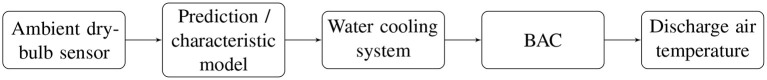
Simplistic process control flow.

The models will be used to predict the water temperature of the BAC based on the ambient temperature and required discharge temperature. This temperature becomes the set-point for the system providing the water temperature at a temperature within 2% of the provided set-point. This process is depicted in Figure 4.

### 2.4. Machine learning prediction methods

Various ML prediction methods exist to characterize and classify systems. However, the temperature set-point needs to be a continuous number and not combined into a range. Classification techniques are therefore not relevant. ML prediction techniques with the ability to do regression characterization will be used in this comparison. The following three commonly used ML regression predictive techniques, identified and described in Section 1, will be compared:

- Artificial Neural Network
- Support Vector Machine
- K-Nearest Neighbors

#### 2.4.1. Artificial neural network

An ANN was developed to represent the BAC and predict the required water temperature for the desired BAC discharge temperature. The ANN was built in Python^®^^2^ using the TensorFlow^3^ library (Van Rossum and Drake, 2009; Abadi et al., 2015). The best hyperparameters were identified using Keras-Tuner^4^ (O'Malley et al., 2019). Table 2 summarizes the hyperparameters of the developed ANN.

**Table 2:** Best ANN hyperparameters identified for the provided dataset.

**Layer number**	**Number of nodes**	**Activation function**
Input layer	2	-
1	7	Scaled Exponential Linear Units (SELU)
2	5	SELU
3	13	SELU
Output layer	1	-

The ANN was trained using mean squared error as a loss metric. An early stop criterion of mean squared error was also used to prevent over-fitting. The data was split into 80% training data and 20% validation data. The training triggered the early stop criterion within 35 epochs and resulted in a mean absolute error of 0.77°C on the validation dataset.

#### 2.4.2. Support vector machines

The regression model of the SVM algorithm (SVR) in the Scikit-Learn^5^ library was used to form the SVM prediction method (Pedregosa et al., 2011). The input and output data are scaled using a the standard scaler from Scikit-Learn and the regression model provides a scaled output. The output is inversely scaled to obtain the prediction value for the water temperature. The GridSearchCV function (from Scikit-Learn Pedregosa et al., 2011) was used to obtain the optimal parameters for the prediction model. The optimal parameters are listed in Table 3.

**Table 3:** Best SVM parameters identified for the provided dataset.

**Parameter**	**Best value**	**Parameter**
	**or function**	**description**
Kernel	Poly	Kernel function type
*C*	10	Regularization parameter
γ	Scale	Kernel coefficient
*coef*0	0.01	Kernel projection coefficient
Degree	3	Degree of polynomial kernel function


Similarly, the data was split into 80% training data and 20% validation data. Using the optimal parameters in Table 3, the RMSE for testing data was 0.72°C.

#### 2.4.3. K-Nearest neighbor

Multivariable k-NN regression was implemented to characterize the BAC. The mean value of the k nearest points is the output of the regression. For the BAC characterization, the 2 input variables form the coordinates to determine the proximity to the desired point and mean of the corresponding output variables forms the result of the regression.

**Figure 5:**
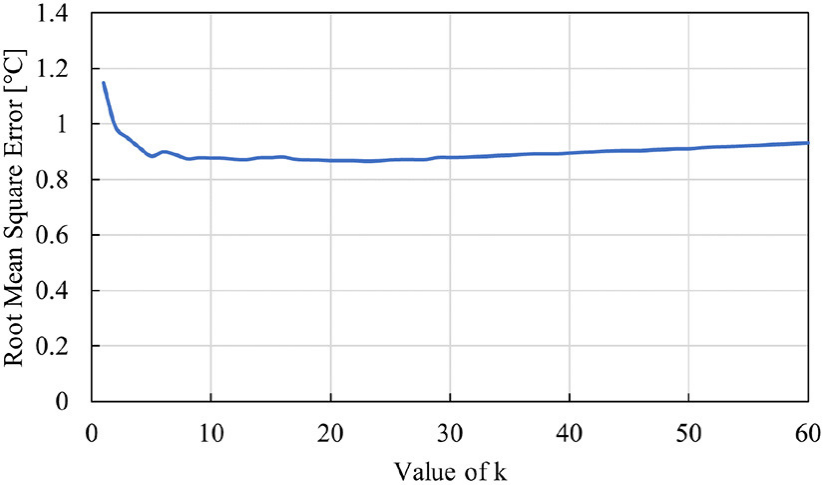
Comparison of RMSE for various values of k.

The k-NN was developed in python using the Scikit-Learn library (Pedregosa et al., 2011). The filtered data was divided into a validation and test dataset according to random 80–20% split, respectively. The value of k (number of nearest neighbors) was identified through evaluating the RMSE (Root-Mean-Square Error) on the test dataset for values of k ranging from 1 to 60. These values are depicted in Figure 5.

The optimal value for k was determined to be 23 with a RMSE of 0.87°C. This k value was used in the model for predicting the water temperature and thus in the comparison to the other two ML prediction methods.

### 2.5. Comparison of techniques

The various models are verified through simulation. A calibrated simulation built in Process Toolbox is used to verify the predictions of the various models. Process Toolbox is a transient thermohydraulic simulation software predominantly used in the mining industry (Maré, 2017). The various methods will be compared according to:

1. Accuracy on a day's simulated ambient temperature profile
2. Data required to obtain a 5% average error on an unseen dataset
3. Time required to train the models and make predictions within the control environment (implemented on the computers available at the respective mine)

The best model (based on the requirements above) is then selected for validation through implementation. The model will predict the water temperature required based on actual present ambient conditions and the discharge air temperatures will be compared to the desired set-points. The power consumption for the entire refrigeration system will be recorded and compared to similar ambient temperature days to evaluate the cost and power saving benefit achieved by the prediction model.

## 3. Results and discussion

**Figure 6:**
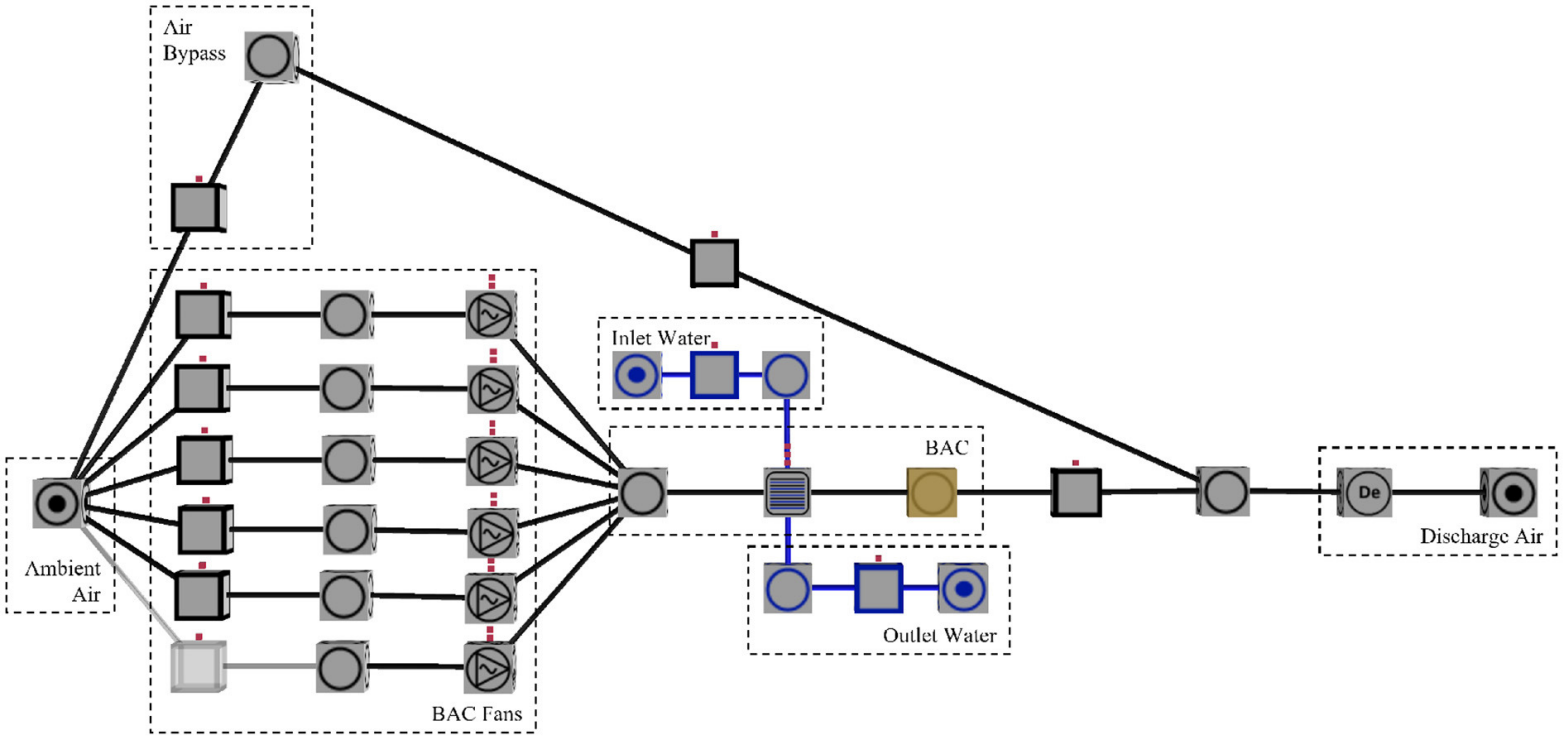
Calibrated process toolbox simulation built for ML prediction model validation.

The design specifications were used to build the BAC simulation model in Process Toolbox. Process Toolbox is a transient thermal hydraulic software widely used for verification and scenario simulation in the mining industry (Maré, 2017; Mathews et al., 2020; Harmse et al., 2022). Operating data along with manual measurements were used to calibrate the simulation within 1% error. The water temperature, ambient temperature, and humidity are inputs, while the simulation provides a discharge air temperature as an output. The built and calibrated simulation can be seen in Figure 6.

As depicted in Figure 6, the ambient air either passes through the BAC or bypasses the BAC to supply the demand. However, the BAC fans ensure that 90% of the air supplied to the demand passes through the BAC. Cool water, with a temperature determined by the water set-point, is supplied to the BAC to cool the air passing through, resulting in the desired discharge air temperature.

### 3.1. Accuracy

**Figure 7:**
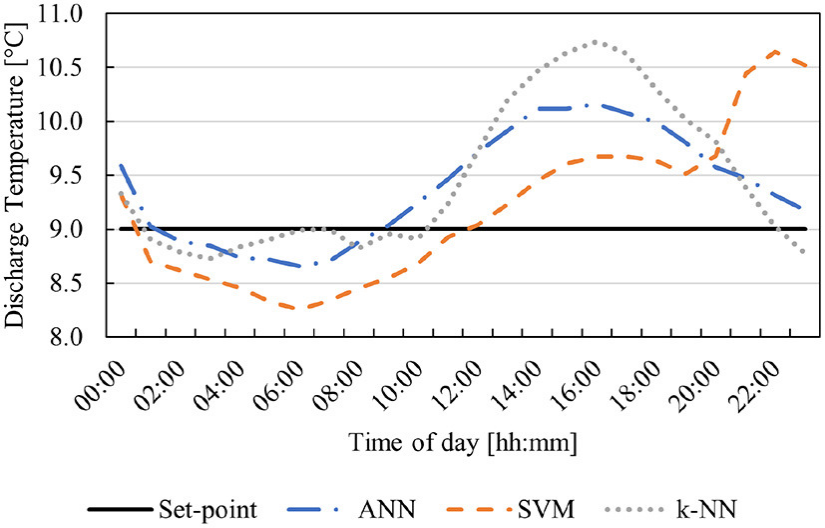
Comparison of ML predictive models for an average day's ambient conditions.

**Figure 8:**
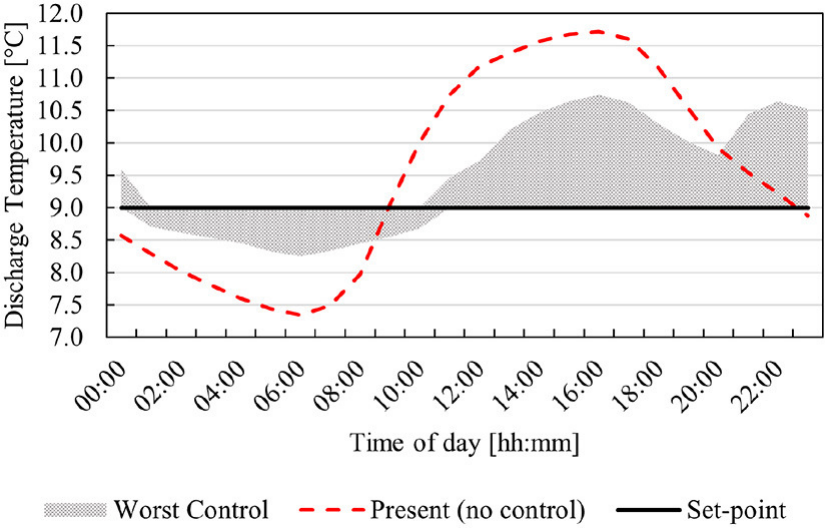
Comparison of worst simulated control- and present discharge temperature for an average day's ambient conditions.

An average day profile of the ambient temperature and humidity is provided to the various models as an input to predict the required water temperature to ensure a discharge temperature of 9°C. The water temperatures provided from the various models are then simulated and the discharge air temperature is compared to the discharge set-point (9°C). The resulting discharge temperature profiles are depicted in Figure 7. Further, the simulated temperatures for the model that is furthest from the set-point at each time period are compared to the present discharge temperature in Figure 8.

As seen in Figure 7, the SVM method resulted in the lowest minimum discharge temperature. The resulting discharge profile varies around 9°C with an average discharge temperature of 9.13°C. The prediction model is susceptible to inaccuracy for areas within the modeling space with less data points (as seen at the end of the profile). This is acceptable, as the thermal properties of the mine will smooth the profile out over the course of a day. Thus, the working areas deeper in the mine will experience a fairly stable temperature comparable to a consistent 9°C discharge temperature.

The ANN and k-NN methods follow similar profiles. The average temperature for the ANN is 9.38°C. This means that the working areas will experience a temperature slightly higher than that of a consistent 9°C discharge temperature. The k-NN method has the highest average temperature (average of 9.46°C) and the highest maximum temperature. This will result in the highest experienced temperature at the working areas. Compared to the present conditions with no control, all three methods provide a more stable BAC discharge temperature. A more detailed comparison of the various methods can be seen in Table 4.

**Table 4:** Accuracy comparison of predictive methods.

Parameter	ANN	SVM	k-NN
Average temperature error [°C]	0.38	0.18	0.46
Average percentage error [%]	4.26	1.97	5.14
Mean percentage absolute error [%]	5.72	6.75	6.42
Maximum percentage absolute error [%]	12.90	18.24	19.34

*The highlighted values indicate the best values for those parameters*.

The highlighted cells in Table 4 indicate the most accurate result, which indicates that the SVM prediction model provided the best overall accuracy. The SVM prediction model has the closest average to the set-point and the lowest maximum error. This means that the working areas will experience the least deviation from a consistent 9°C discharge temperature compared to the other two models, with the k-NN resulting in the largest deviation experienced.

### 3.2. Data points required for 5% average error

The training and predicting process was iterated for the various models to determine the amount of data points required for an average error of below 5%. The predictions were implemented on an daily profile dataset that has not yet been used in the model, with the discharge temperature set-point remaining 9°C. The predicted water temperatures were simulated to determine the discharge air temperature and then the average error can be calculated. This iterative process continued until an average error of 5% was achieved. The required data points for the various models to achieve an average error of 5% can be seen in Table 5.

**Table 5:** Data points required to obtain a 5% average error.

**Model**	**Number of**	**Percentage of**
	**data points**	**entire dataset (%)**
ANN	1,448	87.5
SVM	1,299	78.5
k-NN	N/A	N/A

*The highlighted values indicate the best values for those parameters*.

The k-NN prediction model was not able to achieve 5% average error for the entire dataset, consisting of 1 655 data points. The amount of data points required for 5% average error is thus unknown. The SVM prediction model required the least amount of data points to achieve 5% average error. This is expected seeing as the average error for the entire dataset was the lowest. However, the SVM achieved a 5% average error with 9% less data points than the ANN.

### 3.3. Training and prediction time

The time taken for training the various models excludes obtaining, loading and filtering the data. This is because it will result in similar times across the various models. The prediction time entails predicting the required water temperature for a day's 24 point average profile. Similarly, the time taken for predicting excludes loading the day's average data. The recorded training, predicting and total time can be seen in Table 6.

**Table 6:** Time comparison of predictive methods.

**Parameter**	**ANN**	**SVM**	**k-NN**
Training time [s]	2.5000	0.3429	-
Prediction time [s]	0.2509	0.0309	0.0014
Total time [s]	2.7509	0.3738	0.0014

*The highlighted values indicate the best values for those parameters*.

Due to the nature of the k-NN method, no training time is required, only loading the filtered data and predicting the outcome. The k-NN prediction model had the lowest times for predicting and total time. These results are contrary to common understanding but similar results have been documented in similar comparisons (Colas and Brazdil, 2006; Li et al., 2017). However, the time constant of the system (the BAC and refrigeration system) is in the order of 30 min. This means that even-though the ANN had the worst training, predicting and total times, it is sufficient to be implemented in the control philosophy.

### 3.4. Comparison summary

The SVM prediction model resulted in the best average accuracy. However, an unevenly spread dataset resulted in a large maximum prediction error. This is not an issue for the ventilation system (due to the thermal properties) but is not desirable for the other mining systems, such as the compressed air and dewatering systems that do not have the large filter effect.

The ANN prediction model resulted in the most robust prediction (an acceptable average error with the lowest maximum prediction error). However, the training and prediction time was the longest of the three methods. This is not a concern for the mining industry as all of the systems have time constants in the vicinity of minutes and as a result, a prediction time within seconds is negligible.

The k-NN prediction model was not able to achieve an average error of 5% with the provided dataset. The k-NN prediction model did exhibit the lowest training and prediction time. However, this is not necessary for the mining industry due to the large time constants of the various systems (ventilation and refrigeration, compressed air and dewatering).

Incorporating all the factors, the various methods can be ranked based on the time constants of the respective system. The mining systems can largely be divided into two groups:

1. Large time constants (larger than 2 h)
  - Ventilation system
2. Small time constants (less than 2 h)
  - Compressed air system
  - Dewatering system

The above groupings are a guide as the type of application and can reduce and extend the time constants. Thus, the time constants should be measured and the respective group can then be determined. Once the group is determined, the adequate prediction model can be chosen according to Table 7. The ranking in Table 7 is based on a uniform dataset. If the dataset is not uniform, the ANN prediction model is most applicable due to it resulting in the most robust prediction results.

**Table 7:** Ranking of prediction models based on application.

**Ranking**	**Large time**	**Small time**
	**constants (**>**2 h)**	**constant (**<**2 h)**
1	SVM	ANN
2	ANN	SVM
3	k-NN	k-NN

### 3.5. Simulation implementation

The ventilation system is characterized by a time constant larger than 2 h and as a result, the SVM prediction method is more suitable. The theoretical impact of the prediction model can be obtained through simulation. The water temperature is provided to the refrigeration system and the power consumption required is simulated. This is then compared to the present power consumption. As a result, the prediction model is able to reduce the power consumption on average by 215 kW. This is applicable to the summer which is equivalent to R1.33-million per annum ($82 900) (see text footnote ^1^).

## 4. Conclusion

The deep-level mining industry lacks integrated dynamic control. There are numerous factors that contribute to the lack of dynamic control, with the largest contributor being a lack of sensor equipment and data. The use of ML prediction techniques can be used in integrated dynamic control systems with limited data and sensor equipment, and was identified as a possible solution that can be implemented in the deep-level mining industry.

This study utilized various ML prediction techniques (namely ANN, SVM and k-NN) to predict the water temperature required to achieve a desired discharge temperature of a Bulk Air Cooler supplying chilled air underground to a deep-level mine. The application also serves as basis for comparison of these techniques within the deep-level mining industry by using the specific mine as a case study. It is expected that the methodology will yield similar results in other deep-level mines as well, seeing as it was developed to be a generically adaptable method to address the lack of integrated dynamic control in the deep-level mining environment.

For the case study mine, the SVM prediction model achieved the lowest average error (1.97%) but also exhibited a large maximum error. This is not a concern for the ventilation system which acts as a large filter with the large time constant. The slowest training and prediction time was achieved by the ANN prediction model (total of 2.75 s) which is more than sufficient in the deep-level mining industry with the smallest time constants in the vicinity of minutes. The ANN prediction model achieved the most robust predictions for the water temperature due to the data-points not being evenly distributed across the modeling space.

The SVM prediction was more suitable for the ventilation system due to the large time constant of the system. The implementation thereof could result in an energy saving of 215 kW or 2.9% of the ventilation and refrigeration system, equivalent to R1.33-million per annum ($82 900) (see text footnote ^1^).

Future research is required to determine the strategy for implementation and the sustainability thereof in the unique deep-level mining environment. It is also proposed that the strategy be implemented on other mines to confirm that the results are similar to the results obtained from implementing the strategy at the case study mine, as expected. Further, it is suggested that future work evaluates the impact of data features on the accuracy of the prediction models to determine the best accuracy for capital cost. The comparison of the training of the prediction times can also be further evaluated to determine the variation in results due to dataset features and dataset size.

## Data availability statement

The datasets presented in this article are not readily available because confidentiality from the respective mine prevents sharing of original data. Requests to access the datasets should be directed to MH, mdharmse@gmail.com.

## Author contributions

MH and WP contributed to conception and design of the study. MH developed the models and simulation and wrote the first draft of the manuscript. JL wrote sections of the manuscript. CS provided technical inputs and key insights into the engineering aspects. All authors contributed to manuscript revision, read, and approved the submitted version.

## Funding

The work was sponsored by Integrated Post Graduate Industry Partnership (IPGIP) NPC, South Africa. ETA Operations is an industrial partner of IPGIP.

## Conflict of interest

The authors declare that the research was conducted in the absence of any commercial or financial relationships that could be construed as a potential conflict of interest.

## Publisher's note

All claims expressed in this article are solely those of the authors and do not necessarily represent those of their affiliated organizations, or those of the publisher, the editors and the reviewers. Any product that may be evaluated in this article, or claim that may be made by its manufacturer, is not guaranteed or endorsed by the publisher.

## Footnotes

^1^Exchange: $1 = R16.05 on 6 May 2022.

^2^Python is an open-source object-oriented programming language.

^3^The TensorFlow library is an open-source library (for Python) for machine learning, optimized for neural networks.

^4^The Keras-Tuner library is an open-source library (for Python) for identifying the optimal hyperparamters for a defined dataset and model.

^5^The Scikit-Learn library is an open-source library (for Python) for machine learning containing various models and functions for the optimisation of these models.
